# Internet-Based Cognitive Behavioral Therapy for Patients With Chronic Somatic Conditions: A Meta-Analytic Review

**DOI:** 10.2196/jmir.2777

**Published:** 2014-03-27

**Authors:** Sylvia van Beugen, Maaike Ferwerda, Dane Hoeve, Maroeska M Rovers, Saskia Spillekom-van Koulil, Henriët van Middendorp, Andrea WM Evers

**Affiliations:** ^1^Leiden UniversityInstitute of Psychology, Health, Medical and Neuropsychology UnitLeidenNetherlands; ^2^Radboud University Medical CenterDepartment of Medical PsychologyNijmegenNetherlands; ^3^Radboud University Medical CenterDepartment for Health EvidenceNijmegenNetherlands

**Keywords:** eHealth, internet, intervention, self-management, cognitive behavior therapy, meta-analysis

## Abstract

**Background:**

Patients with chronic somatic conditions face unique challenges accessing mental health care outside of their homes due to symptoms and physical limitations. Internet-based cognitive behavioral therapy (ICBT) has shown to be effective for various psychological conditions. The increasing number of recent trials need to be systematically evaluated and quantitatively analyzed to determine whether ICBT is also effective for chronic somatic conditions and to gain insight into the types of problems that could be targeted.

**Objective:**

Our goal was to describe and evaluate the effectiveness of guided ICBT interventions for chronic somatic conditions on general psychological outcomes, disease-related physical outcomes, and disease-related impact on daily life outcomes. The role of treatment length was also examined.

**Methods:**

PubMed, PsycINFO, and Embase were searched from inception until February 2012, by combining search terms indicative of effect studies, Internet, and cognitive behavioral therapy. Studies were included if they fulfilled the following six criteria: (1) randomized controlled trial, (2) Internet-based interventions, (3) based on cognitive behavioral therapy, (4) therapist-guided, (5) adult (≥18 years old) patients with an existing chronic somatic condition, and (6) published in English. 23 randomized controlled trials of guided ICBT were selected by 2 independent raters after reviewing 4848 abstracts. Demographic, clinical, and methodological variables were extracted. Standardized mean differences were calculated between intervention and control conditions for each outcome and pooled using random effects models when appropriate.

**Results:**

Guided ICBT was shown to improve all outcome categories with small effect sizes for generic psychological outcomes (effect size range 0.17-0.21) and occasionally larger effects for disease-specific physical outcomes (effect size range 0.07 to 1.19) and disease-related impact outcomes (effect size range 0.17-1.11). Interventions with a longer treatment duration (>6 weeks) led to more consistent effects on depression.

**Conclusions:**

Guided ICBT appears to be a promising and effective treatment for chronic somatic conditions to improve psychological and physical functioning and disease-related impact. The most consistent improvements were found for disease-specific outcomes, which supports the possible relevance of tailoring interventions to specific patient groups. Explorative analyses revealed that longer treatment length holds the promise of larger treatment effects for the specific outcome of depression. While the current meta-analysis focused on several chronic somatic conditions, future meta-analyses for separate chronic somatic conditions can further consolidate these results, also in terms of cost-effectiveness.

## Introduction

Cognitive behavioral therapy (CBT) focuses on challenging cognitive distortions and dysfunctional underlying beliefs, and on teaching coping and problem solving skills [1]. A variety of techniques are combined to achieve this, including cognitive restructuring, relaxation, problem solving, and stress management. The central idea of CBT is that the way people make sense of their environment affects their feelings and behavior. CBT is an extensively researched and widely used form of treatment for a variety of psychological conditions [1] and is increasingly used to help a growing number of patients suffering from chronic somatic conditions cope with the consequences of their condition [1-5]. CBT models can, for instance, be applied to improve patients’ adjustment to receiving a diagnosis of a chronic somatic condition and coping with it, to improve comorbid mood problems such as anxiety and depression, to alter disease-specific beliefs and attitudes, and to teach pain/symptom management strategies [6,7].

Although studies indicate that CBT may be an effective treatment for chronic somatic conditions, it has not been implemented on a large scale, partly due to the lack of CBT therapists specializing in patients with chronic somatic conditions. Furthermore, chronically ill patients may have physical limitations that make it difficult to travel to a clinic for face-to-face CBT. A possible solution is to offer CBT online: Internet-based cognitive behavioral therapy (ICBT). Generally, ICBT takes the form of an online self-help program, guided by a therapist who gives feedback and answers questions [8]. Advantages of ICBT over offline computerized CBT and over bibliotherapy include the possibility of the patient connecting with a therapist or with peers who cope with similar problems, and the ability to log on and use the intervention anytime and anywhere they would like*.* ICBT may be beneficial to both patients and therapists: it is more convenient, flexible, and reduces traveling time, costs, and waiting lists, enabling more patients to be reached and treated [9]. In addition, providing CBT online may reduce the stigma of needing psychological help. Recently, first indications have been reported for the cost-effectiveness of ICBT [10-12].

Internet interventions are generally found to be effective for a variety of psychological conditions [13-16]. Preliminary evidence is also emerging for its effect on psychological and physical outcomes in various health problems [17-21] and in promoting health behavior change [22,23]. In order to determine whether ICBT is effective for chronic somatic conditions, the results of the increasing number of recent randomized controlled trials (RCTs) need to be systematically evaluated and quantitatively analyzed. Moreover, knowledge of which types of outcomes are specifically improved by ICBT will provide insight into the types of problems that could be targeted with ICBT.

An additional focus on which elements of interventions are effective for which patients at what disease stage will aid development of effective tailored interventions. Scarce evidence suggests that the amount of therapist contact is related to effectiveness [16]. An aspect of ICBT that has not been examined is whether the duration of ICBT influences treatment outcomes. For traditional face-to-face CBT for chronic somatic conditions, an average treatment of 12-16 sessions given once a week is suggested [24]. Although there are indications in patients with depressive symptoms that a longer ICBT treatment duration yields better outcomes [25], the role of treatment duration has not yet been examined for chronic somatic conditions.

The current review aims to describe and evaluate the effectiveness of guided ICBT interventions in randomized controlled trials, for three specific outcome categories—general psychological outcomes, disease-related physical outcomes, and disease-related impact outcomes—and to explore the role of treatment duration*.* The review focused on guided ICBT interventions, in order to optimize comparability with face-to-face CBT and decrease heterogeneity, as it is known that guided ICBT interventions generally lead to different (larger) effects than non-guided self-help interventions [16]. This review has a broad focus, including a large population of chronic somatic conditions. Because the literature on ICBT in different chronic somatic conditions is rather limited at this time, it is not yet possible to meaningfully summarize the evidence for efficacy of ICBT for these separate categories of chronic somatic conditions. Because the main elements of CBT are generic in scope and can be applied to a large variety of problems, combining these different chronic somatic conditions in this meta-analysis provides a first overall indication of the efficacy of ICBT interventions in the large population of chronic somatic conditions. In addition, the separate outcomes for different somatic conditions can also be deduced from the paper.

## Methods

### Search Strategy and Inclusion Criteria

**Figure 1:**
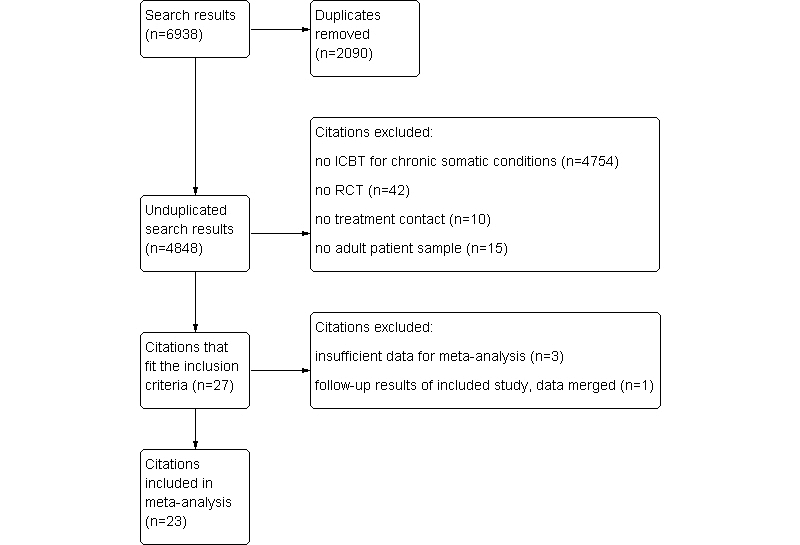
PRISMA flow diagram of study selection.

PubMed, PsycINFO, and Embase were searched from inception until February 2012, by combining index terms indicative of effect studies, Internet, and cognitive behavior therapy, and including the following Medical Subject Heading (MeSH) terms: Internet, electronic mail, behavior therapy, psychotherapy, rehabilitation, counseling, and self-care (see Multimedia Appendix 1 for search strategies). Only studies investigating guided ICBT, which is comparable to face-to-face CBT, were included. All retrieved references were loaded into Endnote, and 2 raters (SvB, MSc Psychology, HvM, PhD Psychology) independently screened titles and abstracts without blinding to authorship or journal. The full text of potentially relevant studies was examined. Discrepancies between reviewers were resolved by discussion. The kappa statistic was calculated to determine consistency among raters. Inclusion criteria were (1) RCT or equivalence trial, (2) therapy provided with the Internet (not face-to-face, telephone, onsite computerized therapy, videoconferencing, or personal digital assistants) as the main way of communication (eg, patient spends >50% of total intervention time spent on an Internet-based intervention), (3) therapy based on CBT principles (in which at least some forms of cognitive and behavioral techniques are used), (4) therapy guided by contact with a therapist, with at least one episode of personalized patient contact (either through asynchronous messages, telephone, or another mode of contact), and (5) adult study sample (age ≥18 years) with an existing chronic somatic condition (ie, a condition expected to last a year or longer, limit what a patient can do, and/or may require ongoing medical care) [26]. Etiology was not an inclusion criterion; both functional and structural disorders were included. Conditions that may have physical consequences but do not have physical illness as its primary feature, such as eating disorders, insomnia, addiction problems, fertility problems, and sexual dysfunction, were also excluded. Papers not published in English were also excluded. Studies were excluded when the main focus of the intervention was focused on lifestyle change, such as increasing levels of exercise or improving diet. Publications of the same intervention were included if each study was based on a new patient sample. Papers were excluded based on a hierarchical approach, in which articles were not further assessed for remaining reasons if they were excluded based on a previous reason. The hierarchy of reasons for exclusion were that (1) the study does not examine ICBT for chronic somatic conditions, (2) the study is not an RCT, (3) the ICBT intervention is not guided by a therapist, and (4) the study does not examine adult patient populations (see Figure 1).

### Data Extraction

The following information was gathered per study: publication year, chronic somatic condition, country of data collection, number of patients included, completers, dropouts, dropout reasons, age, gender, type of CBT intervention, therapist contact, control condition, outcome measures, intervention length, completer or intent-to-treat analyses, post-treatment results, and follow-up results. A large variety of outcome measures were reported across studies. To enable general conclusions, these were grouped together into three main outcome categories that are of relevance to patients with chronic somatic conditions: (1) general psychological outcomes of depression, anxiety, and distress, (2) disease-related physical outcomes related to symptom severity, such as pain, fatigue, and headache, and (3) disease-related outcomes concerning the impact of a chronic somatic condition on daily life (ie, disease-specific distress and disease-specific quality of life) (see Multimedia Appendix 2). To improve homogeneity and narrow the scope of the review, outcome measures that did not fit these categories (eg, coping or behavior) or that were not suitable for pooling in meta-analysis (ie, because of being assessed infrequently (eg, general quality of life) or by means of different measures (eg, disability) were excluded. When more than one outcome was used to measure the same construct, results for the outcome that was most generic (eg, total scale score versus subscale scores), most validated (eg, Beck Depression Inventory (BDI [27]) versus Modified Beck Depression Inventory (mBDI [28]), or most comparable to other studies (eg, visual analogue scale [VAS] of distress versus therapist-rated distress) was used, to prevent separate studies having too much influence on the analysis.

### Assessment of Risk of Bias in Included Studies

Two independent authors (SvB, MSc Psychology, MF, MSc Psychology) assessed each study using the Cochrane risk of bias tool, including selection bias (randomization process), performance bias (blinding of subjects and personnel), detection bias (blinding of outcome assessment), reporting bias (handling of missing data), and attrition bias (reasons for withdrawal in all conditions) [29]. A third rater (MR, professor of evidence-based surgery) was consulted to reach consensus when 2 raters were in disagreement. Risk of bias was assessed based on the information of original publications and on trial registrations on the ClinicalTrials website.

### Reporting Study Results

Only between-group results were taken into account to examine the effect of ICBT as compared to a passive control condition. Passive control conditions were defined as conditions in which participants do not receive a therapeutic program and instead are placed on a waiting list, or receive only treatment as usual or treatment that is theorized to not lead to changes in therapeutic outcomes (eg, patient education) (see Multimedia Appendix 2). For equivalence trials, in which patients receive an intervention that is theorized to lead to clinically relevant changes in outcomes as an active comparison condition, and for studies with a three-arm design, both between-group effects and main effects are reported (see Multimedia Appendices 3 and 4). Intent-to-treat analyses (ITT), in which all randomized patients are analyzed regardless of adherence to study protocol [30], were used wherever possible. When two active ICBT interventions were compared to a passive control condition in a three-arm RCT design, both comparisons are reported. Two types of dropout rates were calculated: (1) intervention dropouts by dividing the number of patients reported to have stopped the intervention (or did not return post-intervention questionnaires) by the number randomized to the intervention group, and (2) measurement dropouts by dividing the number of patients from both the intervention and control groups who did not return post-intervention questionnaires by the total number of patients randomized. As between-group follow-up results were not consistently and uniformly reported across studies, pooling was not feasible. Therefore, only post-intervention study results are reported and the number of studies that included follow-up results are briefly summarized.

### Data Analyses and Synthesis

Standardized mean difference of effect sizes (SMDs) were calculated by subtracting the difference in means in the ICBT group from the difference in means in the control group and dividing the outcome by their pooled standard deviation [31]. Effect sizes of 0.2, 0.5, and 0.8 can be considered as small, moderate, and large, respectively [32]. When a study contained multiple eligible ICBT treatment groups, these were combined in a single pairwise comparison, according to recommendations and calculation methods from the Cochrane handbook [29]. If mean values and SDs were not reported, authors were contacted to obtain original trial data. When not provided, alternative methods were used (ie, using reported mean change scores and associated SDs). To decide whether meta-analytic pooling of data was justified, we computed *I* ^2^, which describes the percentage of total variation between studies due to heterogeneity rather than chance [33]. An *I* ^2^of 25%, 50%, and 75% can tentatively be considered as low, moderate, and high heterogeneity, respectively [33]. High heterogeneity indicates that the effects are not the same for all studies and that there may be other variables that explain this heterogeneity. As significant heterogeneity is to be expected, SMDs were calculated in random effects models, using Cochrane Collaboration software Review Manager, version 5.1. These models assume that there is no one “true effect size”, but rather the effect sizes are sampled from a population of varying effect sizes [34]. Subgroup differences in intervention duration were analyzed using the chi-square test, with *P*<.05 indicating statistically significant differences.

## Results

### Search Results and Study Characteristics

The literature search identified 4848 unique studies, 23 of which met the inclusion criteria (see Figure 1) [35-57]. Interrater reliability of study selection was kappa=.805. The included studies involved 4340 subjects (2299 ICBT and 2041 control); 59% of subjects participated in three large studies by Lorig and colleagues [52-54].

In 74% (17/23) of studies, subjects were randomized to one of two conditions, 15 of which compared ICBT with a passive control condition: waiting-list (12 studies), care-as-usual (2 studies), and information-based psycho-education (1 study) (Multimedia Appendix 2). Three studies compared ICBT with an active CBT control condition: face-to-face group therapy, online stress management without CBT, and ICBT with added telephone contact (Multimedia Appendix 3). Five studies used a three-arm design, two of which reported results of the two joint intervention groups compared to a passive control condition (Multimedia Appendix 2), and three compared each of the three conditions (Multimedia Appendix 4).

A total of 70% (16/23) of studies were published between 2008 and 2011, and 52% (12/23) were carried out in Sweden. Eleven studies (48%) used intent-to-treat (ITT) analyses. The majority of these studies (6/11) used the last observation carried forward (LOCF) method, in which a participant’s missing values after dropout are replaced with the last available measurement [58]. Four of the 11 studies used mixed models approaches [59], and 1 used multiple imputation by chained equations [60]. 74% (17/23) included some form of follow-up assessment ranging from 1-18 months: 10 (43%) used a between-group follow-up and 7 (30%) included a within-group or completers-only follow-up, ranging from 2 months to 1 year. Dropout rates differed widely but were overall relatively high (median 18%, range 2-57%), particularly in the intervention groups (median 29%, range 1-72%) (Multimedia Appendix 2). Of the 5 studies that reported reasons for dropout, the most common reason mentioned was lack of time.

### Patient Populations

Patient populations included chronic pain (5/23 studies, 21%), headache or migraine (4/23 studies, 17%), tinnitus (4/23 studies, 17%), irritable bowel syndrome (IBS, 4/23 studies, 17%), diabetes (2/23 studies, 8%), breast cancer (1/23 studies, 4%), epilepsy (1/23 studies, 4%), fatigue in patients with chronic neurological disorders (1/23 studies, 4%), and a heterogeneous patient population (1/23 studies, 4%) (Multimedia Appendix 2). Twenty studies of 23 (87%) involved community-based samples. The mean age range of subjects within studies varied between 34 and 66 years; most studies included more female than male subjects.

### Intervention Content and Duration

Interventions consisted of a variety of generic CBT-based techniques, often supplemented with specific approaches appropriate for the chronic condition under study. Interventions focusing on relaxation and psycho-education were included only when combined with other CBT techniques, that is, some form of cognitive reappraisal or restructuring [61]. Treatment content was categorized into well-known CBT elements such as cognitive therapy, behavioral therapy, applied relaxation, and psycho-education (see Multimedia Appendix 2). The vast majority of studies described the interventions as self-help programs with structured modules, which were typically completed in a rate of one module per week, with minimal therapist guidance. Interventions consisted of a variety of generic CBT-based techniques, often supplemented with specific approaches appropriate for the chronic condition under study. The most commonly mentioned intervention components were cognitive therapy techniques, (applied) relaxation, psycho-education, and improving coping skills. These components were mentioned in 74-100% of interventions. Stress management and behavioral therapy techniques were also mentioned in over half of included interventions. Other therapy components, incorporated in 26-35% of interventions, were problem solving techniques, mindfulness-based techniques, exposure, and physical exercise. The majority of interventions were labeled as CBT and/or self-management interventions, while some interventions were based on acceptance and commitment therapy (ACT) [46], exposure-based treatment in combination with mindfulness techniques [49-51], or mindfulness-based cognitive therapy (MBCT) [56].

Interventions were generally broad and multifaceted, targeting various aspects of chronic somatic conditions within one intervention (eg, comorbid mental health problems, coping with the chronic somatic condition, and reducing physical symptoms). Incidentally, studies indicated that there was a specific primary aim, for example, to reduce depressive symptoms [56-57], distress associated with the condition [35,37], or severity of the chronic somatic condition [41,43,50]. However, also in the interventions with a more specific aim, components were generally included to fit other aims as well. Therefore, it was not possible to meaningfully categorize interventions according to the intervention aim (eg, physical, mental, prevention). When analyzing the results, the SMDs in each meta-analysis did not meaningfully differ from one another, indicating that there are no differences in SMDs according to intervention aim.

### Therapist Contact and Peer Contact

All studies incorporated treatment-related contact options, usually in the form of (weekly) email contact with (psychology master students supervised by) licensed clinical psychologists. One study was based solely on therapist-patient contact via email without additional treatment components. Most studies did not report, or not in detail, the average time therapists spent on patients. The main mode of therapist contact was through asynchronous (email) messages, but in 3 of 23 studies (13%) telephone was the main contact option. Five studies (22%) used online group formats. A total of 43% (10/23) of studies included a bulletin board that enabled patients to interact with each other, as an addition to individual treatment tools.

### Risk of Bias in Included Studies

**Figure 2:**
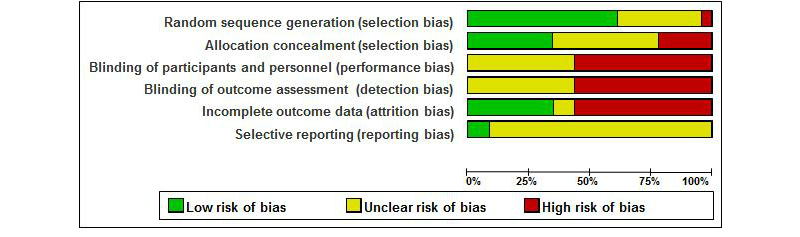
Risk of bias graph.

**Figure 3:**
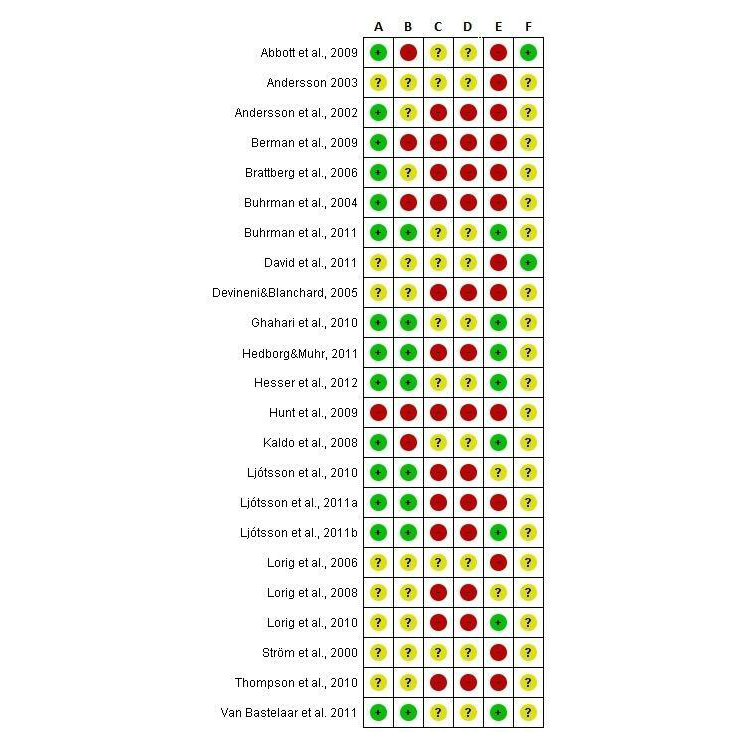
Risk of bias summary: review authors' judgements for each included study about each risk of bias item. A=Random sequence generation (selection bias); B=Allocation concealment (selection bias); C=Blinding of participants and personnel (performance bias); D=Blinding of outcome assessment (detection bias); E=Incomplete outcome data (attrition bias); F=Selective reporting (reporting bias).

The authors’ judgments about risk of bias for each included study and presented as percentages across all included studies can be found in Figures 2 and 3. While the majority of studies (14/23, 61%) reported adequate methods of randomization, 35% (8/23) of studies did not report randomization methods, and 4% (1/23) reported inadequate methods. The study with inadequate methods (eg, randomization based on order of enrollment [47]) was excluded from primary analyses, as a randomized design was one of the inclusion criteria for this study. To be complete, we also report the results including this study, in a secondary analysis. In 8 studies of the 23 (35%), allocation of participants was adequately concealed, while allocation concealment remained unclear in 10 of 23 studies (43%) and was at risk for inadequate concealment in 22% (5/23); for example, tossing a coin, picking a piece of paper, or throwing dice. None of the included studies reported blinding of participants, personnel, and outcome assessments, which led to an unclear risk of bias in 43% of studies (10/23; no information on blinding) or a high risk of bias in 57% of studies (13/23; information indicating that blinding did not take place). Over half of all studies had incomplete outcome data that led to a high risk of bias, which was mainly due to a lack of intent-to-treat analyses in 48% (11/23) of studies. The risk of selective reporting bias remained largely unclear, mainly because only 26% (6/23) were registered with the ClinicalTrials site and registration often took place after study completion.

### Effectiveness of ICBT Interventions

SMDs for the included outcomes are reported in Multimedia Appendix 2 for the 17 studies with a passive control condition, Multimedia Appendix 3 for the 3 studies with an active control condition, and Multimedia Appendix 4 for the 3 studies with a three-arm design. Pooled SMDs for the three outcome categories can be found in Table 1.

**Table 1:** Pooled SMDs for ICBT versus passive control conditions.

Outcome category		*k* ^a^	SMD^b^	95% CI	*z*	*P*	*I* ^2^(%)
**General psychological outcomes**							
	Depressive symptoms	15	0.21	0.08-0.34	3.18	.001	29
	Anxious symptoms	10	0.17	0.01-0.32	2.14	.03	0
	General distress	6	0.21	0.00-0.41	1.98	.05	0
**Disease-related physical outcomes**							
	Irritable bowel syndrome (IBS) symptoms	2	1.19	0.82-1.57	6.25	<.001	0
	Headache	3	0.49	0.21-0.77	3.41	<.001	0
	Sleep quality	3	0.25	-0.02 to 0.53	1.80	.07	0
	Pain	6	0.18	0.08-0.28	3.61	<.001	0
	Fatigue	2	0.15	0.05-0.26	2.87	<.01	0
	Tinnitus loudness	2	-0.04	-0.40 to 0.32	0.24	.81	0
	Glycemic control	2	0.07	-0.17 to 0.30	0.54	.59	62
**Disease-related impact outcomes**							
	Disease-specific quality of life	3	1.11	0.79-1.44	6.73	<.001	0
	Disease-specific distress	6	0.17	0.03-0.31	2.41	.02	57

^a^ *k*=number of comparisons.

^b^SMD=standardized mean difference.

### General Psychological Outcomes

**Figure 4:**
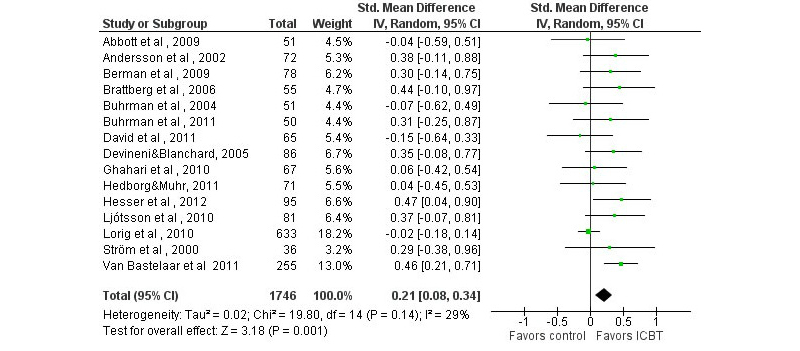
Forest plot of standardized mean differences of the effect on depression of Internet-based cognitive behavioral therapy compared with a passive control condition.

**Figure 5:**
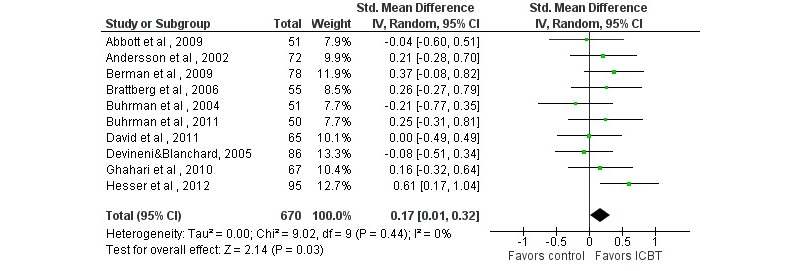
Forest plot of standardized mean differences of the effect on anxiety of Internet-based cognitive behavioral therapy compared with a passive control condition.

**Figure 6:**
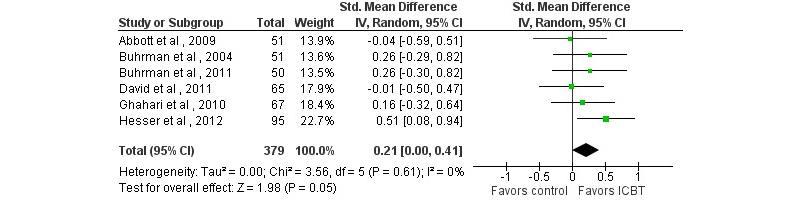
Forest plot of standardized mean differences of the effect on general distress of Internet-based cognitive behavioral therapy compared with a passive control condition.

Sixteen of 17 studies comparing ICBT with a passive control condition included general psychological outcomes, 5 of which (31%) found greater improvements in the ICBT condition on at least one outcome (see Multimedia Appendices 2 and 4). ICBT had similar effects as active treatment control conditions (see Multimedia Appendices 3 and 4). Pooled SMDs for depressive symptoms, anxious symptoms, and general distress yielded small but generally statistically significant effects (see Table 1 and Figures 4 to 6). For depressive symptoms, results of a sensitivity analysis excluding one outlier with a very large effect on depression (SMD 4.34, [56]) are reported; if included, the SMD would be 0.32 (*k*=16, 95% CI 0.09-0.55, *P*=.005, *I* ^2^=78%).

### Disease-Related Physical Outcomes

Seventeen studies comparing ICBT with a passive control condition included disease-related physical outcomes, with 59% (10/17) finding effects in favor of the ICBT condition on at least one outcome (see Multimedia Appendices 2 and 4). Pooled SMDs for physical outcomes yielded varying results. Large effects were found for IBS symptoms, moderate effects for headache, small effects for pain and fatigue, and non-significant effects were found for tinnitus loudness, sleep quality, and glycemic control (see Table 1). In the case of IBS symptoms, one study was excluded based on inadequate randomization procedures. A secondary sensitivity analysis including this study led to very similar results as the primary analysis (pooled SMD 1.14, 95% CI 0.81-1.48, *P*<.001, *I* ^2^=0%, *k*=3). Studies with an active control condition were not pooled due to a limited number of studies and comparable outcomes (see Multimedia Appendices 3 and 4 for the results of individual studies).

### Disease-Related Impact on Daily Life

**Figure 7:**
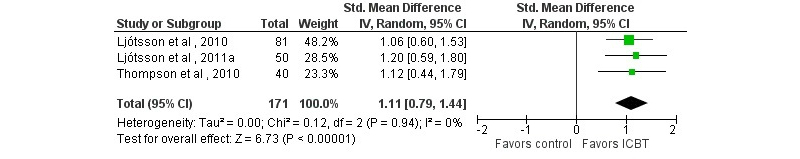
Forest plot of standardized mean differences of the effect on disease-specific quality of life of Internet-based cognitive behavioral therapy compared with a passive control condition.

**Figure 8:**
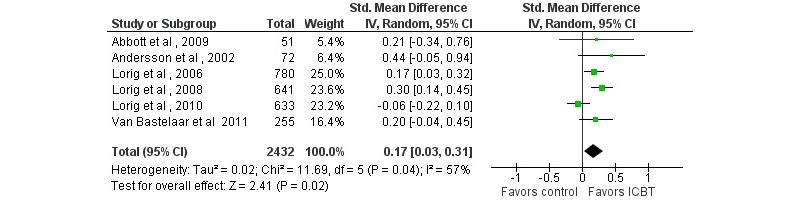
Forest plot of standardized mean differences of the effect on disease-specific distress of Internet-based cognitive behavioral therapy compared with a passive control condition.

Nine studies with a passive control condition included measures of disease-related distress or quality of life, of which 7 (78%) found effects in favor of the ICBT condition on at least one outcome (see Multimedia Appendices 2 and 4). Small but significant effects were found on disease-related distress, and large effects were found on disease-specific quality of life (see Table 1 and Figures 7 and 8). In the case of disease-specific quality of life, one study was excluded based on inadequate randomization procedures. A secondary sensitivity analysis including this study led to very similar results as the primary analysis (pooled SMD 1.09, 95% CI 0.80-1.39, *P*<.001, *I* ^2^=0%, *k*=4). Results from studies with an active control condition were not pooled due to a limited number of studies and outcomes. Individual study results can be found in Multimedia Appendices 3 and 4.

### Role of Treatment Duration on Intervention Effectiveness

Most interventions were relatively short, with little variability in treatment duration: 4% (1/23) of the interventions lasted 4 weeks, 48% (11/23) lasted 6 weeks, and 48% (11/23) lasted 7-24 weeks (see Multimedia Appendix 2). Consequently, outcomes of the studies in which the intervention lasted ≤6 weeks and >6 weeks were compared. Of the 5 studies finding a between-group effect on depression, 4 (80%) had an intervention duration of >6 weeks. Effect sizes of the longer interventions (n=8; SMD 0.29; 95% CI 0.13-0.46) were larger than those in the shorter interventions, with marginal statistical significance (n=7; SMD 0.08; 95% CI -0.05 to 0.22) (χ^2^_1_=3.91, *P*=.05). Intervention duration did not influence effectiveness for other outcomes.

## Discussion

### Principal Findings

Our meta-analysis indicates that ICBT is effective for chronic somatic conditions regarding both general psychological outcomes and disease-specific outcomes. Effect sizes were generally small to moderate, with larger effect sizes occasionally found for disease-related outcomes, such as self-reported headache and IBS symptoms, and for disease-specific quality of life. These findings of larger effects on disease-specific outcomes may on the one hand reflect the larger sensitivity to change of these measures [62,63] and on the other hand support the idea of tailoring interventions to the needs of specific patient groups, as disease-specific measures are likely the measures that respond well to more tailored, disease-specific approaches [64-67].

The three included studies that compared ICBT with an active treatment condition showed that ICBT can be as effective as group-based face-to-face CBT, for example. However, two studies also found that ICBT and an informational website without CBT content were similarly effective. These results indicate a need for studies in which the effect of specific components of ICBT are more closely investigated. The role of one such component of ICBT was examined in this meta-analysis—intervention length—suggesting that interventions lasting longer than 6 weeks result in greater improvements in depression.

Overall, results of this review extend previous reviews and meta-analyses, which concluded that ICBT may be a promising adjuvant treatment for psychological outcomes [13-16] and for patients with health problems [17-23]. Meta-analyses have typically reported small [18] to moderate [14,16] pooled effect sizes for Internet-based psychotherapeutic interventions. The results are also comparable to meta-analyses of face-to-face CBT, which typically find small to moderate effect sizes on a variety of outcomes [1,68-70], with sometimes larger disease-specific than more general mood-related effects [69]. Our review adds to previous findings by including all available studies in chronic somatic populations and by identifying differences in effectiveness for specific categories of outcome. With this approach, it was shown for the first time that guided ICBT is effective for various psychological and physical outcomes, with most promising results for disease-related outcomes and that intervention duration might be a determinant of the effectiveness of ICBT for depression. These results underline the potential benefit of ICBT for patients with chronic somatic conditions in helping them cope with the consequences of their condition.

### Limitations

Some potential limitations should be discussed. First, there are still a limited number of studies on ICBT in chronic somatic conditions, and sometimes only one study was available for a specific condition, which precludes drawing reliable conclusions about specific patient groups and generalizing across conditions. Over half of the studies were performed in Sweden by the same authors, but post-hoc analyses did not find differences in outcomes between the Swedish and other studies (data not shown). Women constituted a large proportion of most study populations, reflecting the often unequal gender distribution of different chronic somatic conditions. Second, studies were found to be of variable methodological quality, which may influence both individual study results and overall outcomes in meta-analysis. Although all studies had unclear or high risk of blinding bias, this is often unfeasible or very difficult to achieve in non-pharmacological behavioral interventions and thus may not be a valid indicator of study quality [71]. In many studies, inadequate descriptions resulted in unclear risk of bias. This may be resolved by using guidelines for reporting RCTs [72]. Third, the appropriateness of pooling studies of ICBT for various patient populations can be discussed, as pooling is intended for more or less homogeneous populations and outcomes. The current review included a relatively diverse range of chronic somatic conditions, and outcomes were often assessed with various different questionnaires. However, similar effects and low heterogeneity were found for most outcomes, supporting the idea that the included studies were comparable regarding their outcomes. Including these various studies in this meta-analytic overview provides the reader with a first indication of the overall effectiveness of ICBT for chronic somatic conditions and increases the generalizability of findings [73,74]. As more trials become available in the future, meta-analyses should be performed for separate chronic somatic conditions. Fourth, long-term between-group follow-ups were often lacking, precluding a reliable long-term estimate. Fifth, there was substantial variation in description of treatment content, therapist contact, and dropout. For instance, not all therapist contact was with a trained therapist but could also include “expert” patients, nurses, physicians, occupational therapists, or research assistants. Dropout rates were not always adequately described and generally high, which is a common problem with Internet interventions [75]. Sixth, publication bias cannot be precluded. The current review was limited to published studies, as it was unfeasible to obtain a complete and unbiased overview of all unpublished grey literature on this subject. This may have led to an overestimation of effectiveness, as published studies are generally more likely to include statistically significant results [76]. However, several studies that did not find an effect were included in the current review, indicating that not only studies with significant results are published on this topic.

Finally, we used the pooled standard deviation based on pre- and post-intervention measurements in our meta-analysis. When using change scores in meta-analysis, the most appropriate measure would have been the standard deviation of changes. However, the included studies did not report sufficient information to calculate these standard deviations [29], which has been recognised as a common problem when using change scores. Our approach can, however, be considered as a conservative approach since the calculated standard deviations will be slightly larger than the standard deviations of changes would have been. Another alternative would have been to perform the meta-analysis based on post-intervention measurements, but such an approach does not take into account possible differences in baseline measurements. Nevertheless, we also performed a meta-analysis based on post-intervention measurements results. The results of this meta-analysis were very similar to the change score results reported in our study (data not shown), and would have led to similar conclusions.

### Future Research

Results from this review suggest several areas for future research, related to study methodology and intervention design. More studies with adequate sample sizes focusing on a wider range of chronic somatic conditions with between-group long-term follow-up are needed. Only one study involved older patients [38], yet older patients are often affected by chronic conditions. As dropout is common with ICBT, ways to promote engagement and improve adherence should be investigated. Preliminary research suggests that tailoring interventions may be an effective strategy to promote engagement and adherence [77-79]. Strategies found to be predictive for adherence include increased therapist contact, more frequent website updates, and more frequent intended usage [80]. Also, future research is needed to examine the effects of ICBT on outcomes such as work-related outcomes, health behaviors, and cost-effectiveness, which were not evaluated in this meta-analysis in order to narrow its scope. Last, the “active ingredients” of interventions need to be identified, in order to develop effective interventions for specific problems. Additional control conditions including “sham” treatment websites should be included to assess the specific value of ICBT [81]. Analyses on computer-generated data about how subjects access the website may also be a worthwhile approach to examine engagement, usability, and active ingredients [82].

### Conclusions

The current review indicates that ICBT interventions improve both psychological and disease-related physical outcomes in patients with chronic somatic conditions, with small-to-medium effect sizes. Larger improvements are occasionally found for disease-specific outcomes related to daily-life impact of the illness, which underlines the importance of tailoring interventions to specific (patient) groups. Our results also indicate that interventions of longer duration may be more effective on psychological outcomes such as depression, which implies that tailoring the duration of interventions to specific problems may be appropriate.

## Multimedia Appendix 1

Search strategies for PubMed, PsycINFO, and Embase.

## Multimedia Appendix 2

Study characteristics and post-intervention effects of ICBT for chronic somatic conditions: two-armed studies with a passive control condition.

## Multimedia Appendix 3

Study characteristics and post-intervention effects of ICBT for chronic somatic conditions: two-armed studies with an active comparison condition.

## Multimedia Appendix 4

Study characteristics and between-group post-intervention effects of ICBT for chronic somatic conditions: three-armed studies with two active treatment conditions and one passive control condition.

## Abbreviations

ACT: acceptance and commitment therapy
CBT: cognitive behavioral therapy
CI: confidence interval
IBS: irritable bowel syndrome
ICBT: Internet-based cognitive behavioral therapy
ITT: intent to treat
LOCF: last observation carried forward
MBCT: mindfulness-based cognitive therapy
MeSH: medical subject heading
RCT: randomized controlled trial
SD: standard deviation
SMD: standardized mean difference
