# Supplementary material for: Internet-Based Cognitive Behavioral Therapy for Patients With Chronic Somatic Conditions: A Meta-Analytic Review
Source: J Med Internet Res. 2014 Mar 27;16(3):e88. doi: 10.2196/jmir.2777 (PMC4004147; doi:10.2196/jmir.2777)
Supplement: Supplementary file 1 [file jmir_v16i3e88_app1.pdf]

Multimedia appendix 1. Search strategies.

Pubmed search strategy

("Randomized Controlled Trial"[Publication Type] OR "Randomized Controlled Trials as Topic"[Mesh] OR random*[tiab] OR non-inferiority[tiab] OR equivalen*[tiab] OR rct[tiab] OR single blind[tiab] OR double blind[tiab] OR triple blind[tiab]) AND ("Internet"[Mesh] OR internet*[tiab] OR web*[tiab] OR online[tiab] OR "Therapy, Computer-Assisted"[Mesh] OR ehealth[tiab] OR e-health[tiab] OR "electronic mail"[MeSH Terms] OR "electronic mail"[tiab] OR "e mail"[tiab] OR email[tiab] OR cyber*[tiab] OR icbt[tiab] OR i-cbt[tiab] OR wcbt[tiab] OR w-cbt[tiab]) AND ("Behavior Therapy"[Mesh] OR psychotherapy[mesh] OR "rehabilitation"[Subheading] OR "rehabilitation"[tiab] OR "rehabilitation"[MeSH Terms] OR psychoeducational[tiab] OR psychoeducation[tiab] OR psycho-educational[tiab] OR psycho-education[tiab] OR "Counseling"[Mesh] OR counselling[tiab] OR counseling[tiab] OR ((therapy[tiab] OR therapies[tiab] OR treatment*[tiab]) AND (cognitive[tiab] OR behavior[tiab] OR behavioral[tiab] OR behaviour[tiab] OR behavioural[tiab] OR conditioning[tiab] OR cognition[tiab])) OR "Behavior Therapy"[Mesh] OR behavior modification[tiab] OR behaviour modification[tiab] OR conditioning therapy[tiab] OR conditioning therapies[tiab] OR conditioning treatment[tiab] OR cognition therapy[tiab] OR cognition therapies[tiab] OR cognitive psychotherapy[tiab] OR cognitive psychotherapies[tiab] OR cognitive treatment[tiab] OR "Self Care"[Mesh] OR self care[tiab] OR self help[tiab] OR self management[tiab])

PsycINFO search strategy

((internet* or web* or online* or computer* or electronic* telemedicine or websites or ehealth or e-health or e-mail or email or electronic mail or cyber* or icbt or i-cbt or w-cbt).ti,ab. or (internet or online therapy or telemedicine or computer assisted therapy or computer mediated communication).sh.) AND ((random* or non-inferiority or equivalen* or rct or single blind or double blind or triple blind).ti,ab. OR (("quantitative study" or "empirical study" or follow up study or "treatment outcome/clinical trial").md. AND random*.af) OR exp evidence based practice/ or clinical trials/ or treatment effectiveness evaluation/) AND (exp cognitive behavior therapy/ or exp behavior modification/ or exp behavior therapy/ or exp cognitive restructuring/ or exp cognitive therapy/ or exp dialectical behavior therapy/ or (rehabilitation or psychoeducational or psychoeducation or psycho-educational or psycho-education or counselling or counseling or ((therapy or therapies or treatment*) and (cognitive or behavior or behavioral or behaviour or behavioural or conditioning or cognition)) or behavior modification or behaviour modification or conditioning therapy or conditioning therapies or conditioning treatment or cognition therapy or cognition therapies or cognitive psychotherapy or cognitive psychotherapies or cognitive treatment or self care or self help or self management).ti,ab. or self management/ or exp cognitive therapy/ or exp self monitoring/)

Embase search strategy

((internet* or web* or online* or computer* or electronic* telemedicine or websites or ehealth or e-health or e-mail or email or electronic mail or cyber* or icbt or i-cbt or w-cbt).ti,ab. or (internet or exp telehealth/ or computer assisted therapy or computer assisted therapy).sh.) AND ((random* or non-inferiority or equivalen* or rct or single blind or double blind or triple blind).ti,ab. or (randomized controlled trial or evidence based practice).sh. or (therapy effect.sh. and random*.af.)) AND (exp cognitive therapy/ or exp behavior modification/ or exp behavior therapy/ or exp self care/ or exp self monitoring/ or (rehabilitation or psychoeducational or psychoeducation or psycho-educational or psycho-education or counselling or counseling or ((therapy or therapies or treatment*) and (cognitive or behavior or behavioral or behaviour or behavioural or conditioning or cognition)) or behavior modification or behaviour modification or conditioning therapy or conditioning therapies or conditioning treatment or cognition therapy or cognition therapies or cognitive psychotherapy or cognitive psychotherapies or cognitive treatment or self care or self help or self management).ti,ab.)
