# Supplementary material for: Internet-Based Cognitive Behavioral Therapy for Patients With Chronic Somatic Conditions: A Meta-Analytic Review
Source: J Med Internet Res. 2014 Mar 27;16(3):e88. doi: 10.2196/jmir.2777 (PMC4004147; doi:10.2196/jmir.2777)
Supplement: Supplementary file 2 [file jmir_v16i3e88_app2.pdf]

Multimedia appendix 2. Study characteristics and post-intervention effects of ICBT for chronic somatic conditions: two-armed studies with a passive control condition.

Author, year (population)	Condition, N	Dropout n (%)	Treatment content (duration)	Outcome	Outcome measure[a]	Between group effects[b]
Abbott et al, 2009 [35]<br><br>(tinnitus)	ICBT, 32	Intervention<br>23/32 (72%)	**ICBT (6 wks)**<br>-applied relaxation<br>-cognitive therapy<br>-behavioral therapy<br>-psychoeducation<br>-improving coping skills	**General psychological**		ITT
				depression	DASS-D	0
				anxiety	DASS-A	0
	Online psychoeducation, 24	Measurement<br>32/56 (57%)		stress	DASS-S	0
				**Disease-related physical**		
				tinnitus loudness	VAS	0
				quality of sleep	VAS	0
				**Disease-related impact**		
				tinnitus-related distress and annoyance	TRQ	0
Andersson et al, 2002 [37]<br><br>(tinnitus)	ICBT, 53	Intervention<br>26/53 (49%)	**ICBT (6 wks)**<br>-applied relaxation<br>-cognitive therapy<br>-behavioral therapy<br>-mindfulness & acceptance-based techniques<br>-psychoeducation<br>-improving coping skills	**General psychological**		Non-ITT
				depression	HADS-D	–[e]
				anxiety	HADS-A	–[e]
				**Disease-related physical**		
	Waiting list, 64	Measurement<br>45/117 (38%)		tinnitus loudness	VAS	–[d]
				quality of sleep	VAS	0[c]
				**Disease-related impact**		
				tinnitus-related distress and annoyance	TRQ	–[e]
van Bastelaar et al, 2011 [57]<br><br>(type 1 and 2 diabetes)	ICBT, 125	Intervention<br>72/125 (58%)	**ICBT (8 wks)**<br>-cognitive therapy<br>-applied relaxation<br>-behavioral therapy<br>-stress management<br>-improving coping skills	**General psychological**		ITT
				depression	CES-D	–[f]
				**Disease-related physical**		
	Waiting list, 130	Measurement<br>88/255 (35%)		glycemic control	HbA1c	*n.r.*
				**Disease-related impact**		
				diabetes-specific emotional distress	PAID	–[f]
Berman et al, 2009 [38]<br><br>(chronic pain)	ICBT, 52	Intervention<br>10/52 (19%)	**ICBT (6 wks)**<br>-applied relaxation<br>-cognitive therapy (group)<br>-psychoeducation	**General psychological**		Non-ITT
	Waiting list, 37	Measurement<br>11/89 (12%)		depression	CES-D	0
				anxiety	STAI-6	0
				**Disease-related physical**		
				pain intensity	BPI	0
				**Disease-related impact**		
				–	–	–

Brattberg, 2006 [39]<br><br>(chronic pain and/or burnout)	ICBT, 30	Intervention<br>3/30 (10%)	**ICBT (20 wks)**<br>-cognitive therapy (group)<br>-psychoeducation	**General psychological**		Non-ITT
	Waiting list, 30	Measurement<br>5/60 (8%)		depression	HADS-D	–[d]
				anxiety	HADS-A	0
				**Disease-related physical**		
				bodily pain	SF-36	–[d]
				**Disease-related impact**		
				–	–	–
Burhman et al, 2004 [40]<br><br>(chronic back pain)	ICBT, 22[g]	Intervention<br>*n.r.*	**ICBT (6 wks)**<br>-improving coping skills<br>-applied relaxation<br>-stress management<br>-cognitive therapy<br>-physical exercise<br>-psychoeducation	**General psychological**		Non-ITT
				depression	HADS-D	0
				anxiety	HADS-A	0
	Waiting list, 29[g]	Measurement<br>5/56 (9%)		affective distress	MPI	0
				**Disease-related physical**		
				average pain intensity	Diary	0
				pain severity	MPI	0
				**Disease-related impact**		
				–	–	–
Burhman et al, 2011 [41]<br><br>(chronic back pain)	ICBT, 26	Intervention<br>3/26 (12%)	**ICBT (8 wks)**<br>-applied relaxation<br>-cognitive therapy<br>-stress management<br>-improving coping skills<br>-mindfulness<br>-physical exercise<br>-psychoeducation	**General psychological**		ITT
				depression	HADS-D	0
				anxiety	HADS-A	0
				affective distress	MPI	0
	Waiting list, 28	Measurement<br>4/54 (7%)		**Disease-related physical**		
				pain severity	MPI	0
				**Disease-related impact**		
				–	–	–
David et al, 2011 [42]<br><br>(breast cancer)	ICBT, 69	Intervention<br>37/69 (54%)	**ICBT (8 wks)**<br>-psychoeducation<br>-cognitive therapy<br>-behavioral therapy<br>-stress management<br>-improving coping skills<br>-problem solving	**General psychological**		Non-ITT
				depression	BSI	0
				anxiety	BSI	0
				psychological distress	BSI-GSI	0
	Waiting list, 64	Measurement<br>63/133 (47%)		**Disease-related physical**		
				–	–	–
				**Disease–related impact**		
				–	–	–
Devineni and Blanchard, 2005 [43]<br><br>(chronic headache)	ICBT, 39[g]	Intervention<br>*n.r.*	**ICBT (4 wks)**<br>-applied relaxation<br>-cognitive therapy<br>-stress management<br>-improving coping skills<br>-biofeedback	**General psychological**		Non-ITT
	Waiting list, 47[g]	Measurement<br>53/139 (38%)		depression	CES-D	0
				anxiety	STAI-T	0
				**Disease-related physical**		
				headache index	Diary	–[f]
				**Disease-related impact**		
				–	–	–

Hunt et al, 2009 [47]<br><br>(irritable bowel syndrome)	ICBT, 28	Intervention<br>15/28 (54%)	**ICBT (6 wks)**<br>-applied relaxation<br>-stress management<br>-improving coping skills<br>-cognitive therapy<br>-behavioral therapy<br>-exposure<br>-psychoeducation	**General psychological**		Non-ITT
				–	–	–
				**Disease-related physical**		
	Waiting list (+self-monitoring), 26	Measurement<br>23/54 (43%)		gastrointestinal symptom severity	GSRS	–[e]
				**Disease-related impact**		
				disease-specific quality of life	IBS-QOL	+[d]
Ljótsson et al, 2010 [50]<br><br>(irritable bowel syndrome)	ICBT, 43	Intervention<br>13/43 (30%)	**ICBT (10 wks)**<br>-exposure<br>-mindfulness & acceptance-based techniques<br>-cognitive therapy<br>-behavioral therapy<br>-psychoeducation	**General psychological**		ITT
				depression	MADRS-S	0[h]
				**Disease-related physical**		
	Waiting list +discussion forum, 43	Measurement<br>5/86 (6%)		IBS symptom severity	GSRS-IBS	–[f]
				**Disease-related impact**		
				disease-specific quality of life	IBS-QOL	+[f]
Ljótsson et al, 2011a [49]<br><br>(irritable bowel syndrome)	ICBT, 30	Intervention<br>7/30 (23%)	**ICBT (10 wks)**<br>-exposure<br>-mindfulness & acceptance-based techniques<br>-cognitive therapy<br>-behavioral therapy<br>-psychoeducation	**General psychological**		ITT
				–	–	–
				**Disease-related physical**		
	Waiting list + discussion forum, 31	Measurement<br>11/61 (18%)		IBS symptom severity	GSRS-IBS	–[f]
				**Disease-related impact**		
				disease-specific quality of life	IBS-QOL	+[f]
Lorig et al, 2006 [53]<br><br>(hetero-geneous)	ICBT, 457	Intervention<br>104/457 (23%)	**ICBT (6 wks)**<br>-psychoeducation<br>-physical exercise<br>-cognitive therapy<br>-stress management<br>-improving coping skills<br>-relaxation<br>-problem solving	**General psychological**		Non-ITT
	CAU, 501	Measurement<br>175/958 (18%)		–	–	–
				**Disease-related physical**		
				pain	VNS	–[e]
				fatigue	VNS	–[d]
				**Disease-related impact**		
				health distress	HDS	–[d]
Lorig et al, 2008 [54]<br><br>(arthritis or fibromyalgia)	ICBT, 433	Intervention<br>123/433 (28%)	**ICBT (6 wks)**<br>-psychoeducation<br>-physical exercise<br>-cognitive therapy<br>-applied relaxation<br>-stress management<br>- improving coping skills<br>-problem solving	**General psychological**		ITT
	CAU, 422	Measurement<br>214/855 (25%)		–	–	–
				**Disease-related physical**		
				pain	VNS	–[f]
				fatigue	VNS	0[c]
				**Disease-related impact**		
				health distress	HDS	–[f]

Lorig et al, 2010 [52]<br><br>(type 2 diabetes)	ICBT, 259	Intervention 96/491 (20%)	**ICBT (6 wks)**<br>-stress management<br>-improving coping skills<br>-physical exercise<br>-applied relaxation<br>-problem solving<br>-cognitive therapy<br>-psychoeducation	**General psychological**		ITT
				depression	PHQ	0
	ICBT + email reinforcement, 232	Measurement 116/761 (15%)		**Disease-related physical**		
				glycemic control	A1c	0[c]
	CAU, 270			**Disease-related impact**		
				health distress	HDS	0
Ström et al, 2000 [55]<br><br>(recurrent headache)	ICBT, 20[g]	Intervention<br>*n.r.*	**ICBT (6 wks)**<br>-applied relaxation<br>-problem solving<br>-improving coping skills	**General psychological**		Non-ITT
				depression	BDI	0
				**Disease-related physical**		
	Waiting list, 25[g]	Measurement 57/102 (56%)		headache index	Diary	–[d]
				**Disease-related impact**		
				–	–	–
Thompson et al, 2010 [56]<br><br>(epilepsy)	ICBT, 13	Intervention 7/26 (27%)	**ICBT (8 wks)**<br>-cognitive therapy<br>-mindfulness & acceptance based techniques<br>-psychoeducation<br>-applied relaxation	**General psychological**		Non-ITT
				depression	BDI	–[f]
	MBCT delivered by phone, 12	Measurement 13/52 (25%)		**Disease-related physical**		
				–	–	–
	CAU<br>waiting list, 27			**Disease-related impact**		
				physical health quality of life	BRFSS	0

[a]BDI=Beck Depression Inventory; BPI=Brief Pain Inventory; BRFSS=Behavioral Risk Factor Surveillance System; BSI=Brief Symptom Inventory; BSI-GSI=Brief Symptom Inventory - Global Severity Index; CAU=Care as usual; CES-D=Centre for Epidemiologic Studies – Depression; DASS-A=Depression Anxiety Distress Scales - Anxiety; DASS-D=Depression Anxiety Distress Scales - Depression; DASS-S=Depression Anxiety Distress Scales - Stress; GSRS=Gastrointestinal Symptom Rating Scale; GSRS-IBS=Gastrointestinal Symptom Rating Scale - Irritable Bowel Syndrome; HADS-A=Hospital Anxiety and Depression Scale - Anxiety; HADS-D=Hospital Anxiety and Depression Scale – Depression; HbA1C=Hemoglobin A1C; HDS=Health Distress Scale; IBS=irritable bowel syndrome; IBS-QOL=Irritable Bowel Syndrome Quality of Life Instrument; ICBT=Internet-based cognitive therapy; ITT=intent-to-treat analysis; MADRS-S=Montgomery-Åsberg Depression Rating Scale self-rating; MPI=Multidimensional Pain Inventory; PAID=Problem Areas in Diabetes; PHQ=Patient Health Questionnaire; SF-36=Short Form Health Survey-36; STAI-S=State Trait Anxiety Inventory - State Anxiety; STAI-T=State Trait Anxiety Inventory - Trait Anxiety; TRQ=Tinnitus Reaction Questionnaire; VAS=Visual Analogue Scale; VNS=Visual Numeric Scale.
[b]0 =no statistically significant effects, – =statistically significant effect indicating a reduction in the outcome, + =statistically significant effect showing an increase in the outcome
[c]$P \leq .1$
[d]$P < .05$
[e]$P \leq .01$
[f]$P \leq .001$
[g] after dropout, pre-dropout sample size not reported
[h]Per-protocol means and SDs are reported in the article (ITT did not affect results, except for non-significance of effect on MADRS-S).
