# Supplementary material for: Internet-Based Cognitive Behavioral Therapy for Patients With Chronic Somatic Conditions: A Meta-Analytic Review
Source: J Med Internet Res. 2014 Mar 27;16(3):e88. doi: 10.2196/jmir.2777 (PMC4004147; doi:10.2196/jmir.2777)
Supplement: Supplementary file 3 [file jmir_v16i3e88_app3.pdf]

Multimedia appendix 3. Study characteristics and post-intervention effects of ICBT for chronic somatic conditions: two-armed studies with an active comparison condition.

Author, year (population)	Condition, N	Dropout n (%)	Treatment content (duration)	Outcome	Outcome measure[a]	Main effects ICBT[b]	Between group effects[b]
Andersson et al, 2003 [36]<br><br>(recurrent headache)	ICBT + phone, 24	Intervention 7/24 (29%)	**ICBT (6 wks)**<br>-applied relaxation<br>-problem solving<br>-cognitive therapy<br>-psychoeducation<br>-improving coping skills	**General psychological**		Non-ITT	
				depression	HADS-D	– [c]	0
				anxiety	HADS-A	0	0
				self-perceived stress	PSS	– [d]	0
	ICBT - phone, 20	Measurement 20/44 (45%)		**Disease-related physical**			
				headache index	Diary	0	0
				**Disease-related impact**			
				–	–	–	–
Kaldo et al, 2008 [48]<br><br>(tinnitus)	ICBT, 26	Intervention 10/26 (38%)	**ICBT (6 wks)**<br>-applied relaxation<br>-cognitive therapy<br>-behavioral therapy<br>-exposure<br>-stress management<br>-improving coping skills<br>-psychoeducation<br>-problem solving	**General psychological**		ITT	
				depression	HADS-D	– [g]	0
				anxiety	HADS-A	– [h]	0
	Group-based CBT, 25	Measurement 2/51 (4%)		perceived stress	VAS	0	0
				**Disease-related physical**			
				tinnitus loudness	VAS	– [h]	0
				quality of sleep	ISI	+ [h]	0
				**Disease-related impact**			
				tinnitus-related distress and annoyance	TRQ	– [h]	0

Ljótsson et al, 2011b [51]<br><br>(irritable bowel syndrome)	ICBT, 98	Intervention 1/98 (1%)	**ICBT (10 wks)**<br>-mindfulness & acceptance-based techniques<br>-exposure<br>-behavioral therapy<br>-cognitive therapy	**General psychological**		ITT	
				depression	HADS-D	– [e]	0
				anxiety	HADS-A	– [e]	0
				self-perceived stress	PSS	– [c]	0
	Internet stress management (no exposure content), 97	Measurement 4/195 (2%)	**Stress Management (10 wks)**<br>-stress management<br>-improving coping skills<br>-problem solving<br>-psychoeducation	**Disease-related physical**			
				IBS symptom severity	GSRS-IBS	– [e]	– [e,f]
				**Disease-related impact**			
				disease-specific quality of life	IBS-QOL	+ [e]	– [e,f]

[a]GSRS-IBS=Gastrointestinal Symptom Rating Scale – Irritable Bowel Syndrome; HADS-A=Hospital Anxiety and Depression Scale – Anxiety; HADS-D=Hospital Anxiety and Depression Scale – Depression; IBS-QOL=Irritable Bowel Syndrome Quality of Life Instrument; ICBT = internet-based cognitive behavioral therapy; ISI=Insomnia Severity Index; ITT=intent-to-treat analysis; PSS=Perceived Stress Scale; TRQ=Tinnitus Reaction Questionnaire; VAS=Visual Analogue Scale.
[b]0 =no statistically significant effects, – =statistically significant effect indicating a reduction in the outcome, + =statistically significant effect showing an increase in the outcome.
[c]$P < .05$
[d]$P \leq .01$
[e]$P \leq .001$
[f]Favoring ICBT
[g]Only for the Internet condition
[h]*P* value not reported
