# Supplementary material for: Internet-Based Cognitive Behavioral Therapy for Patients With Chronic Somatic Conditions: A Meta-Analytic Review
Source: J Med Internet Res. 2014 Mar 27;16(3):e88. doi: 10.2196/jmir.2777 (PMC4004147; doi:10.2196/jmir.2777)
Supplement: Supplementary file 4 [file jmir_v16i3e88_app4.pdf]

Multimedia appendix 4. Study characteristics and between-group post-intervention effects of ICBT for chronic somatic conditions: three-armed studies with two active treatment conditions and one passive control condition.

Author, year (population)	Condition, N	Dropout n (%)	Treatment content (duration)	Outcome	Outcome measure[a]	Comparison	Between group effects[b]
Ghahari et al, 2010 [44] (fatigued patients with neurological conditions)	ICBT, 34; Online self-management, 28; Care as usual, 33	Intervention 10/34 (29%)	**ICBT (7 wks)** -stress management -improving coping skills -psychoeducation -cognitive therapy	**General psychological**			ITT
						ICBT - control	0
				depression	DASS-D	Info only - control	0
						ICBT - info only	0
						ICBT - control	0
				anxiety	DASS-A	Info only - control	0
						ICBT - info only	0
		Measurement 10/95 (11%)	**Online self-management (7 wks)** -stress management -improving coping skills -psychoeducation			ICBT - control	0
				stress	DASS-S	Info only - control	0
						ICBT - info only	0
				**Disease-related physical**			
				–	–	–	–
				**Disease-related impact**			
				–	–	–	–
Hedborg & Muhr, 2011 [45] (migraine)	ICBT, 28; ICBT+ massage, 27; Control, 28	Intervention 6/55 (11%)	**ICBT (6 months)** -stress management -improving coping skills -applied relaxation -cognitive therapy -physical exercise -behavioral therapy -psychoeducation	**General psychological**			ITT
						ICBT+ - control	0
				depression	MADRS-S	ICBT - control	0
						ICBT + - ICBT	0
				**Disease-related physical**			
		Measurement 7/83 (8%)				ICBT + - control	–[c]
				migraine frequency	Diary	ICBT - control	–[c]
						ICBT + - ICBT	0
				**Disease-related impact**			
				–	–	–	–

Hesser et al, 2012 [46]<br><br>(tinnitus)	ICBT, 32	Intervention<br>10/67 (15%)	**ICBT (8 wks)**<br>-applied relaxation<br>-cognitive therapy<br>-behavioral therapy<br>-exposure<br>-stress management<br>-improving coping skills<br>-psychoeducation<br>-problem solving	**General psychological**			ITT
						ICBT - control	0
				depression	HADS-D	IACT - control	– [d]
	IACT, 35					ICBT - IACT	0
						ICBT - control	– [d]
				anxiety	HADS-A	IACT - control	– [c]
						ICBT - IACT	0
						ICBT - control	0
	Discussion forum, 32	Measurement<br>4/99 (4%)	**IACT (8 wks)**<br>-mindfulness & acceptance-based techniques<br>-behavioral therapy<br>-cognitive therapy<br>-psychoeducation	stress	PSS	IACT - control	– [d]
						ICBT - IACT	0
				**Disease-related physical**			
						ICBT - control	0
				sleep quality	ISI	IACT - control	0
						ICBT - IACT	0
				**Disease-related impact**			
				–	–	–	–

[a]DASS-A=Depression Anxiety Distress Scales - Anxiety; DASS-D=Depression Anxiety Distress Scales - Depression; DASS-S=Depression Anxiety Distress Scales - Stress; HADS-A=Hospital Anxiety and Depression Scale - Anxiety; HADS-D=Hospital Anxiety and Depression Scale – Depression; IACT=Internet-based acceptance and commitment therapy; ICBT = internet-based cognitive behavioral therapy; ISI=Insomnia Severity Index; ITT=intent-to-treat analysis; MADRS-S=Montgomery-Åsberg Depression Rating Scale self-rating; PSS=Perceived Stress Scale.

[b]0 =no statistically significant effects, – =statistically significant effect indicating a reduction in the outcome, + =statistically significant effect showing an increase in the outcome.

[c]$P<.05$

[d]$P\leq.01$
